# French Medical Students

**Published:** 1847-01

**Authors:** 


*French Medical Students.*—By a decree just issued from the University of France, it has been ordered that, after the 1st of November, 1846, all medical students shall undergo an examination at the end of each of the three years of study. In the first year, on physic, chemistry, and natural history; in the second year, on anatomy and physiology; and in the third year, on pathology. There will be three examiners, and four students at each examination. The examinations will take place between the 15th of July and the 1st of August, and any student who fails to satisfy his examiners, cannot again present himself until the following November. Unless he then passes, his ticket for the ensuing quarter will be withdrawn. If again rejected in November, he must resume his studies for a year.

These rules are severe, but they embody a much better method of testing the knowledge of a candidate than the mere forms of examination which are gone through in this country, upon all kinds of subjects, at one sitting of an hour's duration.—*Med. Gaz.*

